# Detection of genomic mutations in *katG* and *rpoB* genes among multidrug-resistant *Mycobacterium tuberculosis* isolates from Tehran, Iran

**DOI:** 10.1016/j.nmni.2021.100879

**Published:** 2021-04-15

**Authors:** B. Motavaf, N. Keshavarz, F. Ghorbanian, S. Firuzabadi, F. Hosseini, S. Zaker Bostanabad

**Affiliations:** 1) Department of Microbiology, Islamic Azad University, Tehran North Branch, Tehran, Iran; 2) Department of Microbiology, Faculty of Medicine, Islamic Azad University, Tehran Medical Sciences, Tehran, Iran; 3) Microbiology Department, Islamic Azad University-Parand Branch, Tehran, Iran; 4) Mycobacteriology Department, Massoud Laboratory, Tehran, Iran

**Keywords:** *Isoniazid*, *katG*, multidrug-resistant, *Mycobacterium tuberculosis*, *rifampin*, *rpoB*

## Abstract

Multidrug-resistant (MDR) *Mycobacterium tuberculosis* strains, defined as resistant to at least isoniazid and rifampin, have emerged as a major worldwide health threat. Spontaneous point mutations in various genes of *M. tuberculosis* cause resistance to isoniazid, with the most frequent gene target being *katG*; and resistance to rifampin is usually due to mutation in the *rpoB* gene. The current study was aimed to detect the point mutations in the *katG* and *rpoB* regions related to isoniazid and rifampin resistance. A total of 203 respiratory specimens were collected from patients suspected of having tuberculosis respiratory infections referred to hospitals of Tehran, Iran, during 2018–2019. The isolation and identification of *M. tuberculosis* isolates were performed according to the WHO protocol. Drug susceptibility testing was carried out by proportional method. PCR analysis and sequencing were used to detect mutations in the selected *katG* and *rpoB* regions. Forty-four *M. tuberculosis* strains were isolated, of which 12 (27.3%) and 10 (22.7%) were resistant to isoniazid and rifampin, respectively. Ten isolates were resistant to both isoniazid and rifampin and were considered as MDR isolates. Of the ten MDR isolates, six (60%) carried mutations in both *rpoB* and *katG*. The most common mutations among isoniazid- and rifampin-resistant isolates were in codon 315 of the *katG* gene (70%) and codon 441 of the *rpoB* gene (50%), respectively. The results of this study indicated that MDR-TB continues to be a serious public health problem in Iran.

## Introduction

Human tuberculosis (TB), a devastating disease caused by *Mycobacterium tuberculosis*, remains one of the top ten causes of death and the leading cause from a single infectious agent worldwide, with 10 million new cases and 1.4 million deaths in 2019 [1]. The major global concern in the treatment of TB is the emergence of drug-resistant *M. tuberculosis* [2]. Trends indicate that multidrug-resistant (MDR) *M. tuberculosis* strains, defined as simultaneously resistant to the first-line antibiotics isoniazid and rifampin, are spreading rapidly [3]. MDR-TB is considered a major worldwide health threat because treatment of these cases requires second-line anti-TB drugs, which are more toxic, more expensive and still less effective; in addition, the equipment and supplies needed for drug susceptibility testing are not readily available in resource-limited settings [4]. TB treatment success rates of disease caused by MDR isolates of *M. tuberculosis* are worryingly low, with only about half of cases resulting in cure, compared with 80% of drug-susceptible cases [2]. Moreover, MDR-TB is not only a serious epidemiological and clinical issue, but also carries substantial economic costs for the remedy and management of this disease [5].

Early identification of MDR *M. tuberculosis* isolates is a key aspect to reduce and prevent the spread of these resistant strains. Antimicrobial resistance among *M. tuberculosis* strains is principally caused by spontaneous chromosomal mutations in selected genes, low permeability, or efflux pumps. Resistance to first-line anti-TB drugs has been associated with mutations in *katG*, *inhA*, *ahpC* and *kasA* for isoniazid resistance; and *rpoB* for rifampin resistance [6,7]. Investigators from around the world have reported varying geographic distribution of selected mutations of these genes, for instance, the most common mutations detected in *rpoB* of rifampin-resistant strains include codons 531, 526 and 516 [8]. Mutations in various genes are involved in the resistance of *M. tuberculosis* to isoniazid. The most frequently observed were mutations at codon 315, found in more than half of strains [9]. However, resistance to isoniazid can also occur through mutations of *inhA* or its promoter region, which are usually associated with low-level resistance to isoniazid [10,11].

Several molecular approaches rely on these genetic determinants to identify MDR isolates. Importantly, rapid and accurate detection of known mutations for isoniazid and rifampin are comparable to the current reference standard of phenotypic drug susceptibility tests (DST) [12]. Molecular epidemiological studies have previously described the widespread of MDR *M. tuberculosis* strains in Iran. However, the incidence of isoniazid and rifampin resistance and its spread into public health should be monitored constantly in Iran. In the current study, we have investigated the prevalence of *katG* and *rpoB* mutations among MDR *M. tuberculosis* isolates from individuals with TB in Tehran, Iran.

## Materials and methods

### *Mycobacterium tuberculosis* isolates

A total of 203 respiratory specimens (sputum, bronchoalveolar lavage, pleural fluid) were collected from patients suspected of having TB respiratory infection and referred to hospitals of Tehran, Iran, from September 2018 to September 2019. All samples were transferred to the TB reference unit of Masoud Laboratory (under the supervision of the Swedish Institute for Infectious Disease Control). The ethics committee of Islamic Azad University, Tehran North Branch approved this study, and all patients provided written informed consent. The isolation and identification of *M. tuberculosis* isolates were performed according to the WHO protocol [13]. Accordingly, clinical samples were decontaminated using the *N*-acetyl-l-cysteine–sodium hydroxide method. All the smears were stained using Ziehl–Neelsen stain. The samples were cultured in Lowenstein–Jensen medium and the cultures were incubated at 37°C and examined for growth once weekly up to 8 weeks. Identification of *M. tuberculosis* was carried out using the catalase, niacin and nitrate tests, as well as according to the bacterial pigment production and growth rates [13].

### DNA extraction and molecular identification of *M. tuberculosis*

The mycobacterial DNA was extracted using QIAamp® genomic DNA extraction kit (Qiagen, Hilden, Germany) according to the manufacturer's instructions. The quality and quantity of the extracted DNA were evaluated using Nanodrop (DeNovix Inc., Wilmington, DE, USA). Extracted DNA was stored at –20°C for later analysis. In this study, an IS6110-based PCR assay was used to confirm *M. tuberculosis* strains as previously described [14].

### Drug susceptibility testing of *M. tuberculosis*

The DST was carried out by a proportional method as described previously [15]. Resistance was expressed as the percentage of colonies that grew on critical concentrations of the drugs: 0.2 μg/mL for isoniazid and 40 μg/mL for rifampin. *Mycobacterium tuberculosis* H37Rv strain (ATCC 27294) was used for quality control testing in DST.

### PCR amplification and DNA sequencing

A 570-bp fragment of the *rpoB* gene, containing the 81-bp hypervariable region covering positions with the most frequent mutations in rifampin-resistant strains (*rpoB* codons 511, 513, 516, 522, 526, 531 and 533), was amplified using specific primers listed in Table 1. In addition, a 458-bp fragment of *katG* was amplified by PCR with the primers listed in Table 1. The amplification reactions were performed in a final volume of 25 μL, containing 12.5 μl of 2 × Master Mix (Ampliqon, Odense, Denmark), 5 μg of genomic DNA, 10 pmol of each primer and 8.5 μL of distilled water. PCR was performed in an Eppendorf thermal cycler (Eppendorf, Hamburg, Germany), using the following condition: initial denaturation at 94°C for 5 min, followed by 35 cycles of 94°C for 45 seconds, 1 min annealing at specific temperature for each primer set (Table 1), 72°C for 1 min and a final extension step at 72°C for 5 min. PCR amplicons were separate using 1.2% agarose gels and visualized by staining with gel red stain (CinnaGen Co., Tehran, Iran). Finally, the expected amplicon fragments of *katG* and *rpoB* were extracted and purified from agarose gel using Agarose Gel DNA Extraction Kit (Roche Co., Mannheim, Germany) and then sequenced using Bioneer sequencing methods (Bioneer Inc., Daejeon, South Korea). DNA sequences were edited using Chromas Lite version 2.5.1 (Technelysium Pty Ltd, South Brisbane, QLD, Australia). All complete *katG* and *rpoB* nucleotide sequences were aligned to the *katG* and *rpoB* sequences of *M. tuberculosis* H37Rv as a reference sequence using Molecular Evolutionary Genetics Analysis version 7.0 (MEGA7).

**Table 1:** Nucleotide sequences of primers used in PCR and sequencing in this study

Target gene	Oligonucleotide sequence (5ʹ–3ʹ)	Annealing temperature (°C)	PCR product (bp)	Ref.
IS6110	F: CCTGCGAGCGTAGGCGTCGG	68°C	123	[14]
	R: CTCGTCCAGCGCCGCTTCGG			
*katG*	F: CTCGGCGATGAGCGTTAC	57°C	458	[26]
	R: TCCTTGGCGGTGTATTGC			
*rpoB*	F: CGAATATCTGGTCCGCTTG	57°C	570	[26]
	R: GGTCAGGTACACGATCTC			

## Results

Of 203 clinical specimens from individuals with suspected TB, 44 (21.7%) *M. tuberculosis* strains were isolated. According to the sample origin, 38 (86.4%) and 6 (13.6%) *M. tuberculosis* strains were isolated from sputum and bronchoalveolar lavage, respectively. None of the *M. tuberculosis* strains was isolated from pleural fluid samples. Of the 44 patients from whom the *M. tuberculosis* isolates were obtained, 31 (70.4%) were male and 13 (29.6%) were female. The median age of the patients was 42.5 years (range 10.3–82.1 years). Based on the PCR assay using IS6110 for confirmation of *M. tuberculosis* isolates, all isolates showed positive results and the presence of the gene was confirmed by a band at 123 bp.

Phenotypic drug susceptibility to isoniazid and rifampin was assessed for these 44 isolates. Accordingly, 12 (27.3%) and 10 (22.7%) isolates were resistant to isoniazid and rifampin, respectively. In ten (22.7%) cases, isoniazid resistance was combined with resistance to rifampin, and these were considered to be MDR isolates (showing resistance to at least isoniazid and rifampin). Two isolates (4.5%) were monoresistant to isoniazid. Amplification of *katG* and *rpoB* genes in resistant *M. tuberculosis* isolates yielded 458-bp and 570-bp products, respectively. A point mutation at codon 315 of the *katG* gene (AGC > ACC; S→T) was detected in seven out of ten (70%) MDR-resistant isolates. One (10%) MDR-resistant isolate showed a double mutation at codons 315 (AGC > ACC; S→T) and 335 (ATC > GTC; I →V). No mutation was found in the amplified region of *katG* in two MDR isolates, as well as in the two isolates monoresistant to isoniazid. A point mutation at codon 441 of the *rpoB* gene (ACC > TCC; T→S) was the most common mutation among MDR isolates (five out of ten, 50%) followed by mutation at codon 456 (CGG > TGG; R→T) (three out of ten, 30%). No mutation was observed in the amplified region of the *rpoB* gene in two MDR isolates. The distribution of mutations associated with isoniazid and rifampin resistance among *M. tuberculosis* isolates is presented in Table 2.

**Table 2:** Distribution of mutations associated with isoniazid and rifampin resistance among *Mycobacterium tuberculosis* isolates

Isolates	INH	RIF	*katG* mutation			*rpoB* mutation		
			Codon	Nucleotide change	Amino acid change	Codon	Nucleotide change	Amino acid change
Mtb1	R	R	—	—	—	441	ACC > TCC	T→S
Mtb4	R	R	315	AGC > ACC	S→T	—	—	—
Mtb7	R	R	315	AGC > ACC	S→T	441	ACC > TCC	T→S
Mtb12	R	S	—	—	—	—	—	—
Mtb18	R	R	—	—	—	441	ACC > TCC	T→S
Mtb21	R	R	315	AGC > ACC	S→T	456	CGG > TGG	R→T
Mtb22	R	R	315	AGC > ACC	S→T	456	CGG > TGG	R→T
Mtb27	R	R	315	AGC > ACC	S→T	—	—	—
Mtb28	R	S	—	—	—	—	—	—
Mtb32	R	R	315	AGC > ACC	S→T	456	CGG > TGG	R→T
Mtb36	R	R	315,	AGC > ACC	S→T	441	ACC > TCC	T→S
			335	ATC > GTC	I→V			
Mtb42	R	R	315	AGC > ACC	S→T	441	ACC > TCC	T→S

Abbreviations: INH, isoniazid; R, resistant; RIF, rifampin.

## Discussion

Although the incidence of TB has declined in several parts of world, TB drug resistance remains a major public health issue worldwide. Anti-TB drug resistance usually emerges as a result of spontaneous gene mutations in *M. tuberculosis* strains that render the bacteria resistant to the most commonly used antibiotics [16]. Moreover, the emergence and spread of MDR and extensively drug-resistant TB is reaching all corners of the world [17]. Early detection of drug-resistant *M. tuberculosis* strains is crucial to effective patient treatment and successful disease control strategies. In the current study, we investigated resistance to isoniazid and rifampin and also the most common resistance-related mutations among clinical isolates of *M. tuberculosis* in Tehran, Iran.

The relatively high prevalence of MDR-TB (22.7%) compared with previous studies in Iran [18,19], signifies the critical gaps in current TB treatment regimens and control strategies, it requires effort to develop more effective regimens comprising newer drugs that have a distinct mode of action. Previously, Nasiri *et al.* reported that the trend of MDR-TB was significantly increased among previously treated individuals in Iran. Based on their analysis, there was a significant increase in prevalence from 48.0% in 1996 reached 58.0% [20]. The rate of MDR-TB in this study was higher than in previous studies from different cities of Iran, which ranged from 1% to 17% [21,22]. Although Iran has developed various prevention and treatment programmes to reduce the TB burden in recent years, a total of 130 MDR-TB cases have been demonstrated in this country in 2016, according to the WHO report [23].

Previous molecular-based investigations have revealed that resistance to isoniazid and rifampin among *M. tuberculosis* is due to spontaneous chromosomal mutations in specific genomic regions of the related genes [7]. Although the frequencies and patterns of point mutations within *katG* and *rpoB* genes encoding drug resistance to isoniazid and rifampin have been previously reported by various authors in Iran, epidemiological monitoring of the prevalence of these resistance-related mutations should be constantly performed at regular intervals. In this study, nine and eight point mutations were observed in the amplified regions of *katG* and *rpoB* in isoniazid- and rifampin-resistant isolates, respectively. No mutations were identified in the amplified regions of four isoniazid-resistant isolates.

In the present study, 70% of *M. tuberculosis* isolates carried mutations in codon 315 of the *katG* gene, which was approximately in accordance with the data reported by Doustdar., that mutations in the *katG* 315 region were detected in 60% of isoniazid-resistant isolates [24]. In another study, Zakerbostanabad *et al.* reported a high prevalence of mutations in the *katG* 315 (80%) of isoniazid-resistant *M. tuberculosis* isolates [25]. However, these rates are higher than those previously reported by Kardan-Yamchi *et al.* [26]. Overall, a high prevalence of substitutions in codon 315 of *katG* has been observed to occur in regions of Iran with high TB prevalence [27,28]. Similar high proportions of the point mutation in codon 315 of *katG* among isoniazid-resistant *M. tuberculosis* isolates have been described from Turkey (73%) [29], Lithuania (95%) [30], Russia (93.6%) [31] and Kazakhstan (98.4%) [32]. Although point mutations in various genes of *M. tuberculosis* including *katG*, *inhA*, *kasA*, *ndh*, *ahpC* and *mshA* have been described to be associated with isoniazid resistance, the amino acid substitution in codon 315 (AGC > ACC; S→T) in the *katG* gene is suggested to be preferred by the mycobacterium because it reduces activation of isoniazid while maintaining about 40% of the catalase–peroxidase activity required for its virulence [33]. Moreover, point mutations at codon 315 of *katG* have been described previously to confer a range of resistance levels to isoniazid [34]. Therefore, it is worrying to regions where TB is endemic that a high prevalence of this mutation is observed. In our study, four isoniazid-resistant isolates did not carry any mutation in the amplified region of *katG*. It could be due to spontaneous point mutations in other genes including *katG*, *inhA*, *kasA*, *ndh*, *ahpC* and *mshA*, which have been shown to contribute to isoniazid resistance in *M. tuberculosis* [35].

The high frequency of mutation in codons 441 (50%) and 456 (30%) of the *rpoB* gene, which are present in the region called the rifampin-resistance-determining region, among rifampin-resistant isolates in this study was consistent with data reported by Kardan-Yamchi *et al.* in Tehran [26]. In contrast, a study conducted by Tajbakhsh *et al.* in northeast Iran reported that simultaneous mutations were detected in codons 531, 526 and 516 at frequencies of 55.68%, 38.63% and 13.63%, respectively [36]. Phenotypically rifampin-resistant *M. tuberculosis* isolates harboured mutations in *rpoB* codons 516 and 531 were reported at prevalence of 75% and 71% from Russia [37], and at 1.1% and 82.7% from Kazakhstan [32], respectively. Further molecular surveillances are needed in Tehran and indeed other parts of Iran to clarify the most common mutations causing rifampin resistance, to validate the current data and to enable investigation of drug-resistant TB.

In conclusion, the pattern and frequencies of common point mutations conferring resistance to isoniazid and rifampin in *katG* and *rpoB* genomic regions among MDR-TB isolates in Iran were similar to those described previously by others. These data indicated that MDR-TB continues to be a serious public health problem in Iran. Under-reporting and under-diagnosis of MDR-TB, as well as inappropriate treatment regimens, could facilitate the expansion of such drug-resistant *M. tuberculosis*. The high level of association achieved between DST results and molecular assessment of isoniazid and rifampin resistance in this study was remarkable, suggesting that low-cost molecular assays could be effectively used in Iran for the detection of MDR-TB in clinical settings with acceptable sensitivity.

## Conflict of interest

The authors declare that there are no conflicts of interest.

## Funding

None.

## Ethical permissions

The Ethics Committee of Islamic Azad University, Tehran, Iran confirmed the present research.

## Data availability

The data used to support the findings of this study are available from the corresponding author upon request.

## Author contributions

BM contributed to conceptualization methodology and software. NK contributed to data curation and writing the original draft. FG contributed to visualization and investigation. SF contributed to investigation, formal analysis and to writing the original draft. FH contributed to software and validation. SZB was responsible for supervision, project administration, funding acquisition, resources and for reviewing and editing the paper.
